# Harnessing the microbiome for human health

**DOI:** 10.1111/1751-7915.13172

**Published:** 2018-03-26

**Authors:** Shanyn O'Neill, Chuck Young, Josh Grehan

**Affiliations:** ^1^ Finch 200 Inner Belt Rd Somerville MA 02143 USA

The microbiome has vast applications across a broad range of industries – agriculture, beauty, forensics and more. Each of these industries in the emerging field of the microbiome has birthed, and continue to birth, new companies and jobs. Therapeutics developed from the gut microbiome have recently gained popularity and companies in this area have experienced significant growth. One indication of particular interest to these companies is recurrent *Clostridium difficile* (rCDI).

*Clostridium difficile* (CDI) is a bacterial infection that causes diarrhoea, fever, nausea, abdominal pain, and in extreme cases death. CDI has historically been treated with antibiotics. Most CDI patients respond to antibiotic treatment; however, a CDC study released in 2015 showed 20% of patients experience multiple *C. diff* infections. This study also indicated elderly patients are disproportionately affected, with 30% of CDI patients being 65 and older. CDI is particularly lethal for these elderly patients – almost 10% of patients over 65 who contract *C. diff* die within a month (CDC 2015). To provide context as to how menacing this illness is, in the United States, rCDI kills over four times the amount of people HIV does every year (Leesa *et al*., 2015; CDC 2016). With the emergence of antibiotic resistant *C. diff,* patients need an alternative treatment and the gut microbiome offers just that.

Clinical research has shown that altering the gut's bacterial community can treat *C. diff* infections. A study led by Dr. Els van Nood and Sadowsky revealed 15 of 16 patients treated using FMT were cured while the control groups treated with antibiotics saw just 31% and 23% cure rates (van Nood *et al*., 2013). This study is just one of many demonstrating the potential to achieve clinical outcomes using microbial versus antibiotic treatment.

As shown by Dr. van Nood's study, this treatment option has been proven to be so efficacious that the FDA has allowed patients to be treated using this method under enforcement discretion. OpenBiome – a non‐profit stool bank – has provided treatments to over 30 000 rCDI patients under enforcement discretion. As enforcement discretion is a temporary solution, there are multiple companies seeking FDA approval to treat rCDI using microbial therapies. Seres Therapeutics, Vedanta and Finch Therapeutics Group, are some of these companies. Finch, a pioneer in microbial therapies, demonstrates the growth and job creation the gut microbiome sector offers.

Finch's story began almost 30 years ago in 1989 when Tom Borody (a scientific collaborator of Finch) pioneered the modern FMT. Two decades later, in 2011, Alex Khoruts (another scientific collaborator of Finch) created the first universal stool bank in the United States at the University of Minnesota. Mark Smith and James Burgess (cofounders of Finch) built off Dr. Khoruts' model and opened OpenBiome in 2013 as the first public stool bank with a scalable universal donor model in the world.

In 2015, Crestovo and Finch Therapeutics were both formed. Crestovo merged the knowledge and techniques of doctors Borody and Khoruts launching CP101 to treat rCDI. Finch Therapeutics initially launched as a small research team focused on developing targeted therapeutics based on clinical experience with FMT, but entered the race to treat rCDI when the opportunity later presented itself. In the second half of 2017, Finch Therapeutics and Crestovo merged, launching Finch Therapeutics Group, otherwise known as Finch. In under 2 years, Finch grew from two separate companies of less than 10 people each, to over 100 employees.

Many of the same companies who are seeking to treat rCDI using the microbiome are attempting to treat other indications, such as IBD which includes ulcerative colitis (UC). Studies have shown FMT can improve the symptoms of UC and even cause remission. In 2015, a randomized control trial (RCT) by Moayyedi and colleagues revealed seven of nine UC patients treated using the same donor's materials achieved remission (Moayyedi *et al*., 2015). Informed by this, and other studies, companies are building new teams to research and develop treatments for IBD using the microbiome. Finch has partnered with Takeda on this project to accelerate development and grow the IBD team to accommodate this new indication.

The microbiome health field will continue to create jobs as companies mature and new ones are born. With many illnesses beyond rCDI and UC showing positive responses to microbial treatments, this is a nascent field, rich with growth opportunity. As companies add new indications requiring more scientists on their research and development teams, the number of people employed will expand. Beyond research, once companies, such as Finch, receive FDA approval, they will need to grow their production teams –manufacturing, product operations, clinical and quality – to supply enough treatments for patients. With the growth of the production teams, the operations teams will also need to grow to support them.

The microbiome is one of the most exciting new fields in medicine. Millions of patients will eventually benefit from the treatments that are now in development. Many companies such as Finch are eager to bring products to market to help those in need and in the process, employ thousands as they seek to treat various indications, many yet to be discovered. The future is bright for this field of medicine.

## Conflict of interest

The authors of this editorial are employed by Finch Therapeutics Group.
